# Trends in survival and costs in metastatic melanoma in the era of novel targeted and immunotherapeutic drugs

**DOI:** 10.1016/j.esmoop.2021.100320

**Published:** 2021-11-29

**Authors:** M.G. Franken, B. Leeneman, M.J.B. Aarts, A.C.J. van Akkooi, F.W.P.J. van den Berkmortel, M.J. Boers-Sonderen, A.J.M. van den Eertwegh, J.W.B. de Groot, G.A.P. Hospers, E. Kapiteijn, D. Piersma, R.S. van Rijn, K.P.M. Suijkerbuijk, A.A.M. van der Veldt, H.M. Westgeest, M.W.J.M. Wouters, J.B.A.G. Haanen, C.A. Uyl-de Groot

**Affiliations:** 1 Institute for Medical Technology Assessment, Erasmus University Rotterdam, Rotterdam, The Netherlands; 2 Erasmus School of Health Policy and Management, Erasmus University Rotterdam, Rotterdam, The Netherlands; 3 Department of Medical Oncology, Maastricht University Medical Center, Maastricht, The Netherlands; 4 Department of Surgical Oncology, Netherlands Cancer Institute, Antoni van Leeuwenhoek, Amsterdam, The Netherlands; 5 Department of Medical Oncology, Zuyderland Medical Center, Sittard-Geleen, The Netherlands; 6 Department of Medical Oncology, Radboud University Medical Center, Nijmegen, The Netherlands; 7 Department of Medical Oncology, Cancer Center Amsterdam, Amsterdam UMC, Amsterdam, The Netherlands; 8 Oncology Center Isala, Isala, Zwolle, The Netherlands; 9 Department of Medical Oncology, University Medical Center Groningen, Groningen, The Netherlands; 10 Department of Medical Oncology, Leiden University Medical Center, Leiden, The Netherlands; 11 Department of Internal Medicine, Medisch Spectrum Twente, Enschede, The Netherlands; 12 Department of Internal Medicine, Medical Center Leeuwarden, Leeuwarden, The Netherlands; 13 Department of Medical Oncology, UMC Utrecht Cancer Center, Utrecht, The Netherlands; 14 Department of Medical Oncology and Radiology & Nuclear Medicine, Erasmus MC Cancer Institute, Rotterdam, The Netherlands; 15 Department of Internal Medicine, Amphia Hospital, Breda, The Netherlands; 16 Scientific Bureau, Dutch Institute for Clinical Auditing, Leiden, The Netherlands; 17 Department of Biomedical Data Sciences, Leiden University Medical Center, Leiden, The Netherlands; 18 Department of Medical Oncology, Netherlands Cancer Institute, Antoni van Leeuwenhoek, Amsterdam, The Netherlands

**Keywords:** melanoma, survival, costs, targeted drugs, immunotherapeutic drugs

## Abstract

**Background:**

The objective of this study was to evaluate trends in survival and health care costs in metastatic melanoma in the era of targeted and immunotherapeutic drugs.

**Materials and methods:**

Data on survival and health care resource use were retrieved from the Dutch Melanoma Treatment Registry. The Kaplan–Meier method was used to estimate overall survival. Health care costs and budget impact were computed by applying unit costs to individual patient resource use. All outcomes were stratified by year of diagnosis.

**Results:**

Baseline characteristics were balanced across cohort years. The percentage of patients receiving systemic treatment increased from 73% in 2013 to 90% in 2018. Patients received on average 1.85 [standard deviation (SD): 1.14] lines of treatment and 41% of patients received at least two lines of treatment. Median survival increased from 11.8 months in 2013 [95% confidence interval (CI): 10.7-13.7 months] to 21.1 months in 2018 (95% CI: 18.2 months-not reached). Total mean costs were €100 330 (SD: €103 699); systemic treatments accounted for 84% of the total costs. Costs for patients who received systemic treatment [€118 905 (SD: €104 166)] remained reasonably stable over the years even after the introduction of additional (combination of) novel drugs. From mid-2013 to 2018, the total budget impact for all patients was €452.79 million.

**Conclusion:**

Our study shows a gain in survival in the era of novel targeted and immunotherapeutic drugs. These novel drugs came, however, along with substantial health care costs. Further insights into the cost-effectiveness of the novel drugs are crucial for ensuring value for money in the treatment of patients with metastatic melanoma.

## Highlights

• The median survival of patients with metastatic melanoma increased from 11.8 months in 2013 to 21.1 months in 2018.
• The gain in survival came along with substantial health care costs; health care costs were on average €100 300 per patient.
• Costs were much higher for patients with systemic treatment (€118 905) than for patients without systemic treatment (€8316).
• Costs for patients who received systemic treatment remained stable even after the introduction of additional novel drugs.
• Insights into the cost-effectiveness of the novel drugs are crucial for ensuring value for money in metastatic melanoma.

## Introduction

Up until 2011, treatment options were limited for metastatic (unresectable stage IIIc/ stage IV) melanoma and survival was rather poor. Historically, median survival estimates were between 5.5 and 9 months.^1^, ^2^, ^3^, ^4^ In the past decade, however, a myriad of novel immunotherapies [anti-programmed cell death protein 1 (anti-PD-1) and anti-cytotoxic T-lymphocyte-associated protein 4 (CTLA4) antibodies] and targeted drugs [B-type Raf proto-oncogene (*BRAF*) and mitogen-activated protein kinase (*MEK*) inhibitors] became available for metastatic melanoma.^5^ All received market authorisation based on phase III trials and all became, sooner or later, available for Dutch clinical practice.

Real-world evidence showed consistency with the efficacy of the trials^6^, ^7^, ^8^, ^9^ and revealed increased survival over the years in cutaneous melanoma from a median survival of 11.3 months in the period 2013-2014 to 16.9 months in the period 2015-2017.^10^ Although this indicates that the novel drugs are of great value in everyday clinical practice, they come, however, at high acquisition costs. The high costs of novel drugs become increasingly relevant as many patients in clinical practice receive these drugs for a period of up to 2 years (anti-PD-1) or until progression (targeted drugs), receive a combination of two novel drugs, and/or receive different novel drugs consecutively. In a previous study, we showed that health care costs were much higher for patients who received systemic treatment (€105 078) compared to patients who did not receive systemic treatment (€7988).^11^ In the Netherlands, ∼800-900 patients are annually diagnosed with metastatic melanoma and >80% is treated with at least one novel drug.^11^

There is, however, limited evidence to what extent the benefits of these novel drugs outweigh their costs in the real-world clinical setting. Other studies in metastatic melanoma previously reported unfavourable cost-effectiveness ratios up to €312 836 and US$353 993 per quality-adjusted life year (QALY).^12^, ^13^, ^14^ In the context of rising health care expenditures and limited resources, it is crucial that stakeholders are well informed not only on the clinical benefits but also on the health care costs of treating patients with novel cancer drugs in clinical practice. We investigated the trends in terms of gains in survival as well as accrued health care costs for metastatic melanoma in the era of novel targeted and immunotherapeutic drugs.

## Materials and methods

### Patients and data

All data were retrieved from the Dutch Melanoma Treatment Registry (DMTR). The DMTR is a nationwide registry and contains detailed data on all metastatic melanoma patients in the Netherlands including baseline characteristics, treatment, dosages, survival, and hospital resource use. Approval from the medical ethical committee was obtained and the DMTR was not subject to the Medical Research Involving Human Subjects Act. A detailed description of the DMTR has been published elsewhere.^15^ To allow sufficient follow-up, we included all patients (≥18 years of age) diagnosed between July 2013 and December 2018; patients with uveal melanoma were excluded. The data cut-off date was 8 May 2020.

### Resource use and unit costs

As described by the methodology in the Dutch costing manual,^16^ costs were computed by applying unit costs to individual patient resource use. The following resources were included in the cost analysis: systemic treatment, medical imaging, hospital visits, hospital admissions, day-care treatment, surgery, radiotherapy, hyperthermia, radiofrequency ablation (RFA), and genetic testing. Supplementary Table S1, available at https://doi.org/10.1016/j.esmoop.2021.100320, presents the unit costs. Drug costs were retrieved from the Dutch drug database [Z-index^17^; costs excluding value-added tax (VAT)]. Costs of drugs used in clinical trials were set at zero when the type of drug was unknown (i.e. in a blinded trial) or when the drug never received approval for metastatic melanoma. Unit costs of hospital admissions, outpatient visits, and day-care treatment were based on reference prices published in the Dutch costing manual.^16^ Unit costs of medical imaging, surgical procedures, radiotherapy, hyperthermia, RFA, and genetic testing were valued using the tariffs issued by the Dutch Healthcare Authority.^18^ All costs were based on euro 2018 prices; where necessary, costs were adjusted to 2018 prices using the consumer price index from the Dutch Central Bureau of Statistics.^19^

### Data analysis

All outcomes were stratified according to the year of diagnosis of metastatic melanoma. For each diagnosis cohort year, we determined baseline characteristics, type of treatments, overall survival (OS), health care costs (per patient), and budget impact. Descriptive statistics were used to summarize baseline characteristics, type of treatments, and health care costs. Differences in baseline characteristics between cohort years were assessed by one-way analysis of variance for numerical variables and by the chi-square test for categorical variables; distribution of categories was based on known data. The Kaplan–Meier method was used to evaluate median OS and computed from diagnosis of metastatic disease until death or last date of follow-up. The follow-up was based on the absolute total observation period. Total and monthly health care costs were reported in mean, median, and standard deviations (SD) and computed from diagnosis of metastatic disease until death or last date of follow-up (i.e. observation period). Budget impact was computed by summing the costs of all patients. To allow easy interpretation for comparison, budget impact was also standardized for the number of patients in each cohort year (using the average of the last 4 years 2015-2018). All analyses were conducted using STATA statistical analysis software, version 16.1 (StataCorp LLC, College Station, TX).

## Results

### Patient and treatment characteristics

Table 1 shows the baseline patient and tumour characteristics. A total of 4513 patients were diagnosed with metastatic melanoma between July 2013 and December 2018. The baseline characteristics were balanced across cohort years. The mean/median age of all patients was 63 years, most patients were male (59%), had stage IV-M1c disease (69%), an Eastern Cooperative Oncology Group (ECOG) performance status of 0 or 1 (74%), a normal lactate dehydrogenase level (57%), metastases in less than three organ sites (52%), no brain metastases (70%), and *BRAF*-mutated (52%) melanoma.

**Table 1:** Baseline patient and tumour characteristics

	All cohorts	2013	2014	2015	2016	2017	2018	*P* value^a^
Number of patients	4513	410	735	849	818	808	893	
Age, years								**<0.001**^b^
Mean (SD)	63 (13)	60 (14)	63 (13)	63 (13)	64 (14)	63 (14)	65 (13)	
Median (interquartile range)	63 (54-73)	62 (50-70)	65 (55-73)	64 (53-72)	65 (55-73)	66 (55-73)	66 (55-75)	
Sex, *n* (%)								
Male, *n* (%)	2648 (59)	216 (53)	438 (60)	512 (60)	487 (60)	460 (57)	535 (60)	0.117
ECOG performance status, *n* (%)								0.068
0	2032 (45)	190 (46)	324 (44)	423 (50)	354 (43)	361 (45)	380 (43)	
1	1319 (29)	111 (27)	198 (27)	224 (27)	258 (32)	236 (29)	292 (33)	
≥2	591 (13)	45 (11)	92 (13)	105 (12)	115 (14)	117 (14)	117 (13)	
Unknown	571 (13)	64 (16)	121 (16)	97 (11)	91 (11)	94 (12)	104 (12)	
LDH level, *n* (%)								**<0.001**
≤1 ULN	2592 (57)	250 (61)	421 (57)	476 (56)	448 (55)	469 (58)	528 (59)	
>1 ULN to ≤2 ULN	993 (22)	54 (13)	130 (18)	183 (22)	224 (27)	210 (26)	192 (22)	
>2 ULN	570 (13)	56 (14)	94 (13)	118 (14)	82 (10)	93 (12)	127 (14)	
Unknown	358 (8)	50 (12)	90 (12)	72 (8)	64 (8)	36 (4)	46 (5)	
*BRAF* mutation, *n* (%)								0.146
No	1812 (40)	147 (36)	319 (43)	325 (38)	339 (41)	331 (41)	351 (39)	
Yes	2346 (52)	225 (55)	353 (48)	443 (52)	417 (51)	429 (53)	479 (54)	
Unknown	355 (8)	38 (9)	63 (9)	81 (10)	62 (8)	48 (6)	63 (7)	
Disease stage, *n* (%)								**0.040**^c^
M0, M1a, M1b	1062 (24)	90 (22)	151 (21)	189 (22)	197 (24)	184 (23)	251 (28)	
M1c	3118 (69)	270 (66)	505 (69)	592 (70)	564 (68)	590 (73)	597 (67)	
Unknown	333 (7)	50 (12)	79 (11)	68 (8)	57 (7)	34 (4)	45 (5)	
Brain metastases, *n* (%)								0.426
No	3139 (70)	292 (71)	512 (70)	600 (71)	584 (71)	549 (68)	602 (67)	
Yes	1240 (27)	97 (24)	201 (27)	230 (27)	220 (27)	238 (29)	254 (28)	
Unknown	134 (3)	21 (5)	22 (3)	19 (2)	14 (3)	21 (3)	37 (4)	
Distant metastasis, *n* (%)								0.523
<3 organ sites^d^	2360 (52)	210 (51)	372 (51)	462 (54)	432 (53)	414 (51)	470 (53)	
≥3 organ sites	2033 (45)	193 (47)	352 (48)	368 (43)	366 (45)	370 (46)	384 (43)	
Unknown	120 (3)	7 (2)	11 (2)	19 (2)	20 (2)	24 (3)	39 (4)	

*BRAF*, B-type Raf proto-oncogene; ECOG, Eastern Cooperative Oncology Group; LDH, lactate dehydrogenase; SD, standard deviation; ULN, upper limit of normal.

Bold indicates *P*-value <0.05.

a Differences in baseline characteristics between cohort years were assessed by one-way analysis of variance for numerical variables and by the chi-square test for categorical variables; distribution of categories was based on known data.

b Only 2013 statistically significantly differed; *P* value for other years = 0.132.

c Only 2018 statistically significantly differed; *P* value for other years = 0.765.

d Including disease stage M0.

**Figure 1:**
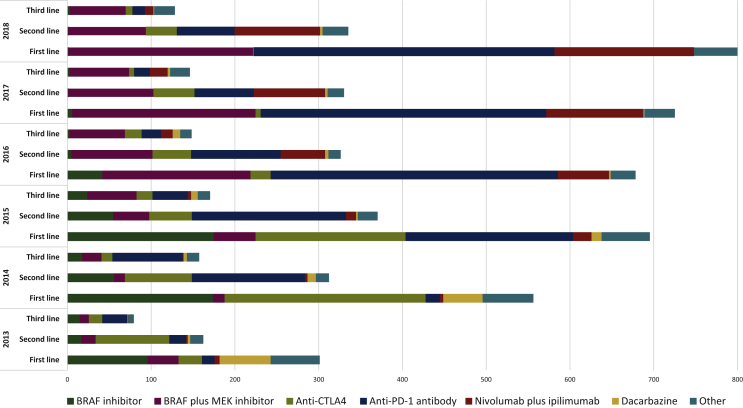
Types of treatment stratified by cohort year and line of treatment. *BRAF*, B-type Raf proto-oncogene; CTLA4, cytotoxic T-lymphocyte-associated protein 4; *MEK*, mitogen-activated protein kinase; PD-1, programmed cell death protein 1.

Most patients (83%) received systemic treatment during our observation period (see Table 2). Over time, however, more patients received systemic treatment, increasing from 73% in 2013 to 90% in 2018. Patients received on average 1.85 (SD: 1.14) lines of systemic treatment. About 41% of patients received at least two lines of treatment, which remained reasonably stable over the years. Supplementary Figure S1, available at https://doi.org/10.1016/j.esmoop.2021.100320, shows the drugs approved over time in the Netherlands. Figure 1 shows the types of drug received by cohort year and by line of treatment. The use of ipilimumab monotherapy and *BRAF* inhibitor monotherapies decreased over the years whereas the use of *BRAF* plus *MEK* inhibitor combination therapy, anti-PD-1 monotherapy, and nivolumab plus ipilimumab increased over the cohort years.

**Table 2:** Treatment characteristics and results of the survival analysis and cost analysis stratified by cohort year

Year of diagnosis	All patients	2013	2014	2015	2016	2017	2018
Number of patients	4513	410	735	849	818	808	893
Received systemic treatment, %	83%	73%	76%	82%	83%	90%	90%
Number of treatment lines, mean (SD)	1.85 (1.14)	2.00 (1.37)	2.03 (1.25)	1.97 (1.23)	1.82 (1.07)	1.76 (1.05)	1.66 (0.96)
Receiving ≥2 lines of treatment, %	41	40	42	44	40	41	38
Observation period in months, mean [median] (SD)	19.7 [14.3] (17.4)	23.7 [11.8] (23.9)	21.7 [11.0] (21.8)	22.8 [13.6] (20.8)	21.2 [17.0] (15.9)	18.3 [18.2] (11.6)	13.1 [14.2] (6.8)
Patients who received systemic treatment, mean	21.2	25.0	24.7	25.5	23.3	19.2	13.8
Patients who did not receive systemic treatment, mean	11.9	20.1	12.1	10.9	10.8	9.8	7.1
Patients alive, %	34	23	19	26	32	42	55
Patients who received systemic treatment, %	37	23	21	28	35	44	58
Patients who did not receive systemic treatment, %	17	25	11	12	17	20	28
Overall survival in months, median (95% CI)	15.6 (14.7-16.7)	11.8 (10.7-13.7)	11.2 (9.8-12.4)	13.8 (11.8-15.5)	17.5 (14.9-19.8)	19.3 (17.2-23.0)	21.1 (18.2-nr)
Patients who received systemic treatment, median (95% CI)	18.8 (17.0-20.0)	13.3 (11.7-15.6)	14.3 (12.4-17.2)	17.0 (15.2-18.9)	20.6 (18.3-24.3)	22.2 (18.9-25.9)	23.2 (20.0-nr)
Patients who did not receive systemic treatment, median (95% CI)	4.1 (3.8-4.6)	5.5 (4.3-9.8)	4.5 (3.8-6.0)	3.7 (3.0-4.6)	3.8 (3.2-4.8)	3.1 (2.4-6.0)	3.0 (2.0-6.0)
One-year survival rate, % (95% CI)	57 (55-58)	50 (45-54)	48 (45-52)	53 (49-56)	60 (57-63)	61 (58-64)	64 (61-67)
Patients who received systemic treatment, % (95% CI)	63 (61-64)	54 (48-60)	56 (52-60)	59 (56-63)	67 (63-70)	65 (61-68)	68 (65-71)
Patients who did not receive systemic treatment, % (95% CI)	28 (24-31)	36 (28-46)	25 (19-31)	23 (17-30)	27 (20-34)	29 (20-39)	30 (21-40)
Two-year survival rate, % (95% CI)	40 (38-41)	33 (29-38)	32 (28-35)	37 (33-40)	42 (38-45)	45 (42-49)	47 (43-52)
Patients who received systemic treatment, % (95% CI)	44 (42-46)	34 (29-39)	38 (34-42)	41 (38-45)	47 (43-50)	48 (44-51)	50 (45-54)
Patients who did not receive systemic treatment, % (95% CI)	20 (17-23)	31 (23-40)	14 (9-19)	14 (9-21)	19 (13-26)	23 (14-32)	27 (19-37)
Three-year survival rate, % (95% CI)	33 (31-34)	30 (24-32)	25 (22-29)	30 (27-33)	34 (31-37)	37 (32-41)	nr
Patients who received systemic treatment, % (95% CI)	36 (34-37)	28 (23-33)	29 (26-33)	34 (30-37)	37 (34-41)	39 (34-44)	nr
Patients who did not receive systemic treatment, % (95% CI)	18 (15-21)	27 (19-36)	13 (8-18)	13 (8-19)	18 (12-25)	19 (11-28)	nr
Costs per patient, mean	€100 330	€93 772	€99 217	€106 177	€105 259	€104 940	€90 014
Costs per patient, median (SD)	€73 656 (€103 669)	€41 798 (€141 848)	€66 235 (€110 681)	€69 993 (€118 385)	€78 950 (€102 997)	€79 309 (€90 552)	€77 314 (€66 309)
Patients who received systemic treatment, mean	€118 905	€124 746	€128 210	€128 203	€125 280	€116 001	€99 390
Patients who did not receive systemic treatment, mean	€8316	€8238	€9160	€6774	€8301	€8318	€9356
Monthly costs per patient, mean (SD)	€6950 (€5684)	€5411 (€4571)	€6223 (€5261)	€6470 (€5522)	€6613 (€5591)	€7485 (€5887)	€8538 (€6118)
Patients who received systemic treatment, mean	€7808	€6616	€7393	€7430	€7415	€7996	€9038
Patients who did not receive systemic treatment, mean	€2701	€2082	€2590	€2138	€2729	€3019	€4245
Budget impact	452.79M	38.45M	72.92M	90.14M	86.10M	84.79M	80.38M
Adjusted budget impact^a^		78.96M	83.54M	89.40M	88.63M	88.36M	75.79M

CI, confidence interval; M, million; nr, not reached; SD, standard deviation.

a Adjusted for number of patients (average of past 4 years).

### Survival

**Figure 2:**
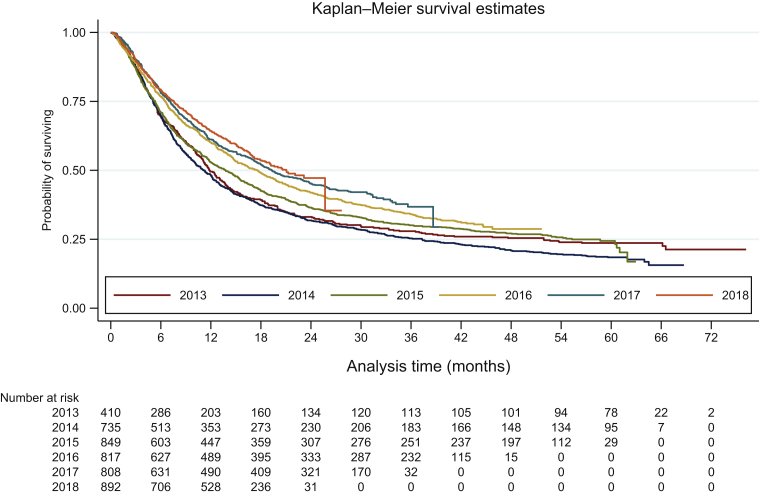
Kaplan–Meier survival estimates stratified by cohort year.

The mean absolute observation period was 19.7 months (see Table 2). In total, 34% of patients were alive at data cut-off. Due to follow-up, more patients were still alive in the last cohort years compared to the first cohort years. Figure 2 presents the Kaplan–Meier estimates by cohort year. The median OS of all patients was 15.6 [95% confidence interval (CI): 14.7-16.7] months. OS, however, increased over the cohort years from 11.8 months in 2013 (95% CI: 10.7-13.7 months) and 11.2 months in 2014 (95% CI: 9.8-12.4 months) to 21.1 months in 2018 (95% CI: 18.2 months-not reached; log-rank test *P* < 0.0001). The 1-year, 2-year, and 3-year survival rates were 57%, 40%, and 33%, respectively (see Table 2). The 1-year survival rate increased from 50% (95% CI: 45% to 54%) in 2013 to 64% (95% CI: 61% to 67%) in 2018 and the 2-year survival rate increased from 33% (95% CI: 29% to 38%) in 2013 to 47% (95% CI: 43% to 52%) in 2018. The increase in 3-year survival rate was smaller, it increased from 30% (95% CI: 24% to 32%) to 37% (95% CI: 32% to 41%) in 2017. Three-year survival rates in cohort 2018 were not yet reached due to follow-up. Median OS and 1-year and 2-year survival rates were statistically significantly better in 2017 and 2018 compared to 2013, 2014, and 2015.

The OS was better in patients who received systemic treatment (OS: 18.8 months; 95% CI: 17.0-20.0 months) compared to patients who did not receive systemic treatment (OS: 4.1 months; 95% CI: 3.8-4.6 months). More importantly, OS for patients who received systemic treatment increased from 9.9 months between 2013 and 2018 [from 13.3 months (95% CI: 11.7-15.6 months) in 2013 to 23.2 months (95% CI: 20.0 months-not reached) in 2018]. Similarly, for survival rates, 1-year and 2-year survival rates of patients who received systemic treatment increased from 54% (95% CI: 48% to 60%) and 33% (95% CI: 29% to 38%) in 2013 to 68% (95% CI: 65% to 71%) and 50% (95% CI: 45% to 54%) in 2018, respectively.

### Health care costs and budget impact

**Figure 3:**
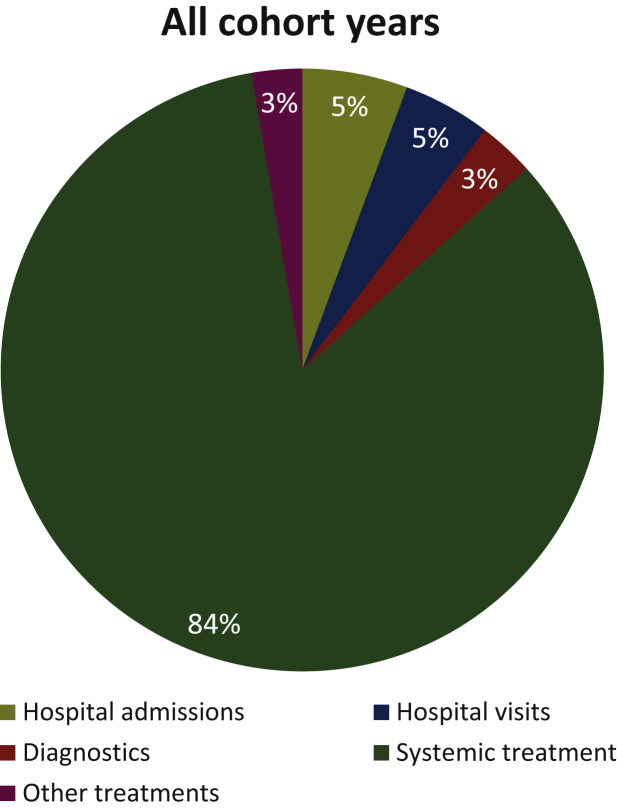
Division of the cost components.

Total mean (median) costs across all cohorts were €100 330 (€73 565; SD: €103 699) per patient (see Table 2). Although total costs increased in the first cohort years from €93 772 in 2013 to €106 177 in 2015, costs were stable in the later cohort years (2016: €105 259 and 2017: €104 940). As expected, costs for patients who received systemic treatment were much higher (€118 905; SD: €104 166) than for patients who did not receive systemic treatment (€8316; SD: €7746). When accounting for differences in follow-up, mean monthly costs remained much higher for patients who received systemic treatment (€7808; SD: €5661) compared to patients who did not receive systemic treatment (€2701; SD: €3447). Figure 3 shows that drug costs were by far the most important cost component accounting for 84% of the total costs. The other costs were accrued by hospital admissions (5%), hospital visits (including day-care treatment; 5%), diagnostics such as medical imaging and genetic testing (3%), and other treatments such as surgery, radiotherapy, hyperthermia, and RFA (3%). The division of the cost components was stable over the years (see Supplementary Figure S2, available at https://doi.org/10.1016/j.esmoop.2021.100320). Further details on costs by line of treatment and by type of treatment have been published by Leeneman et al.^11^

From mid-2013 to 2018, the total budget impact for all 4513 patients was 452.79 million euro (see Table 2). The budget impact per cohort year ranged between 73 and 90 million euro. If adjusted for the number of patients in each cohort, budget impact increased over the years from 79 million euro in 2013 to 89 million and 88 million euro in 2016 and 2017, respectively.

## Discussion

After market approval, novel immunotherapeutic and targeted drugs became the standard of care for metastatic melanoma patients treated in Dutch clinical practice. Using population-based registry data from the nationwide DMTR, we showed that median OS improved from 11.8 months in 2013 to 21.1 months in 2018. The survival curves show a plateau after 4 years. Although this is crucial for patients, our study revealed that the gain in survival in the era of novel immunotherapeutic and targeted drugs came alongside substantial health care costs. The budget impact was ∼70-90 million euro per cohort year. The total estimated budget impact was 452.79 million euro for the treatment of metastatic melanoma patients in the Netherlands during the period mid-2013 to 2018. Costs were on average €100 330 per patient (€118 905 for patients who received systemic treatment and €8316 for patients who did not receive systemic treatment). Drug costs were by far the most important cost driver accounting for 84% of the total costs.

The survival trends observed in Dutch clinical practice are in line with the survival trends of clinical trials over time. For example, the first ipilimumab monotherapy trial showed a median OS of 11.4 months.^20^ Over time, survival increased in clinical trials with *BRAF* inhibitors (median OS: 13.6 months^21^), *BRAF* inhibitor plus *MEK* inhibitor combination therapy (median OS of 22.3^22^ to 26.2 months^23^), anti-PD-1 monotherapy (median OS of 23.5^24^ and 36.9 months^25^), and anti-PD-1 plus anti-CTLA4 (median OS of >60 months^25^).

Although survival trends have been increasing in the era of the novel drugs, it remains somewhat unclear to what extent these increases relate to the survival before the introduction of the novel drugs. A recent systematic literature review^5^ showed that phase III clinical trials between 2010 and 2019 reported survival estimates between 7.4 (95% CI: 6.2-8.7) months^26^ and 13.5 (95% CI: not reported) months^27^ for dacarbazine and other chemotherapies (e.g. temozolomide, paclitaxel). However, the reported OS of the older treatments in these studies was still influenced by the novel drugs as these patients may have received novel drugs after the trial period. Older studies reported survival estimates between 5.5 and 9 months.^1^, ^2^, ^3^, ^4^ In the Netherlands, median survival was 6.4 (95% CI: 5.3-7.7) months between 2003 and 2010 for patients who were diagnosed with synchronous metastatic stage IV melanoma (data of the Netherlands Cancer Registry, see Supplementary Figure S3, available at https://doi.org/10.1016/j.esmoop.2021.100320). The estimates of the Netherlands Cancer registry can, however, not directly be compared to the survival in our study in which all (synchronous and metachronous) metastatic melanoma patients were included. In the DMTR, 83% of the patients had metachronous metastatic disease. These patients had better survival outcomes compared to patients who were diagnosed with synchronous metastatic disease [OS: 16.1 (95% CI: 15.1-17.2) months versus 13.1 (95% CI: 11.2-15.8) months, log-rank *P* = 0.005]; see Supplementary Figure S4, available at https://doi.org/10.1016/j.esmoop.2021.100320.

It is also important to note that survival gains not necessarily arise from the novel treatments. Because of the availability of novel treatments for metastatic disease, it is possible that some of the survival benefits may have been achieved because patients were earlier diagnosed with metastatic disease due to more proactive detection of metastasis. Although it is not possible to distinguish between different causes of the gains in survival, it seems plausible that before the introduction of the novel drugs survival was less favourable than in our first cohort years (2013-2014) in which patients received the first novel drugs.

Similarly, there is limited evidence on the historical costs of metastatic melanoma. As our study shows that ∼84% of the costs were related to drug costs, it is indisputable that health care costs have risen substantially after the introduction of the novel drugs. In a previous study, we showed that monthly health care costs for novel drugs were much higher than the monthly costs for dacarbazine, the historical drug (i.e. monthly costs were 8.4, 5.9, 3.2, and 2.8 times higher compared to dacarbazine for ipilimumab plus nivolumab, *BRAF* plus *MEK* inhibitor, *BRAF* monotherapy, and anti-PD-1 monotherapy, respectively).^11^ Interestingly, our results also show that costs remained reasonably stable over the years, even after the introduction of additional (combination of) novel drugs. It should be noted, however, that we reported costs based on the observation period and total costs will increase as patients continue accruing costs if still alive. This is especially relevant for the last cohorts as more patients are still alive in these cohorts. Moreover, the relation between costs and benefits may differ over time between the novel drugs given that a share of patients live much longer (i.e. the plateau in the survival curves after ∼4 years). According to treatment protocols, some of the novel drugs are given for a limited period (anti-CTLA4 antibodies for four cycles and anti-PD-1s for a maximum of 2 years) whereas other novel drugs are given until progression (*BRAF* and *MEK* inhibitors). A French study extrapolated survival and costs beyond the observation period, which resulted in a mean survival of 23.6 (95% CI: 21.2-26.6) months and mean total costs of €269 682 (95% CI: €244 196-€304 916).^9^ Similarly, a Norwegian health economic study estimated incremental cost-effectiveness ratios ranging between €122 924/QALY (nivolumab compared to dacarbazine) and €312 836/QALY (dabrafenib plus trametinib compared to dacarbazine).^12^ Notwithstanding transferability issues, cost-effectiveness ratios of the novel drugs seem rather unfavourable and above the (informal) threshold in the Netherlands (i.e. €80 000 per QALY for severe diseases). Although it is debatable whether an incremental cost-effectiveness ratio compared to dacarbazine and/or compared to a period before the introduction of novel drugs is still a relevant comparison, it underscores the necessity to increase value for money in metastatic melanoma.

A limitation of our study is that we used list prices for computing drug costs and reference prices and tariffs for other resources, as stipulated by the Dutch costing manual. List prices, reference prices, and tariffs can differ per country. List prices for drugs may also not reflect actual costs as Dutch hospitals negotiate drug prices. Moreover, two drugs (nivolumab and pembrolizumab) are subjected to a confidential financial arrangement at the national level. In 2018, the minister of health reported an aggregated 36% cost reduction for a total of 30 drugs included in financial arrangements.^28^ As negotiated prices and financial arrangements remain confidential, it is not possible to compute actual costs. Lower drug prices, for example, due to price negotiations and/or an increase in the number of indications, are, indisputably, more favourable in terms of value for money and cost-effectiveness. Furthermore, our study only included hospital costs; health care costs outside the hospital and costs outside health care were not included. This implies that total health care and societal costs are higher than our observations. Another limitation could be that the nationwide DMTR only registers patients who are referred to a melanoma centre (i.e. patients with an infaust prognosis not eligible for systemic treatment are not referred to a melanoma centre). This may have introduced some selection bias and overestimated the percentage of patients receiving systemic treatment. The percentage of patients who received systemic treatment increased over the years from 73% in 2013 to 90% in 2018. This could be related to the fact that more novel treatments were introduced over time, but it could also indicate a better selection of patients benefitting from systemic treatment (i.e. no referral to a melanoma centre for patients with an infaust prognosis).

In the context of rising health care expenditures and limited resources, it is important to have insights in the benefits as well as the health care costs of novel immunotherapeutic and targeted drugs in metastatic melanoma. The implementation of a nationwide registry, such as the DMTR, provides such valuable insights into survival and cost outcomes in real-world clinical practice. Our study showed that the gain in survival in the era of novel drugs came along with substantial health care costs. It is questionable to what extent the novel drugs provide value for money given the current cost-effective thresholds. Although data from the DMTR have not been used to influence pricing and reimbursement decision making in the Netherlands, data from nationwide registries can be used for price negotiations to improve value for money in metastatic melanoma. Our results also underline the necessity of a health economic model for further insights into the cost-effectiveness of the novel drugs. In cost-effectiveness analysis, survival outcomes and cost outcomes are extrapolated to a lifetime horizon to include all benefits and costs over time. This would ensure taking into account that a share of patients live much longer (i.e. the plateau in the survival curves after ∼4 years). It would also ensure considering the quality of life of patients. We are currently developing a health economic model including three sequential lines of treatments. This will give insights into the cost-effectiveness of novel drugs as well as the cost-effectiveness of treatment sequences of novel drugs and thus how the novel drugs can be used more cost-effectively in clinical practice. This is crucial for ensuring value for money in the treatment of patients with metastatic melanoma.
