# Supplementary figures and images for: Trends in survival and costs in metastatic melanoma in the era of novel targeted and immunotherapeutic drugs
Source: ESMO Open. 2021 Nov 29;6(6):100320. doi: 10.1016/j.esmoop.2021.100320 (PMC8639434; doi:10.1016/j.esmoop.2021.100320)

**Supplemental Figure A.** Drugs approved for metastatic melanoma

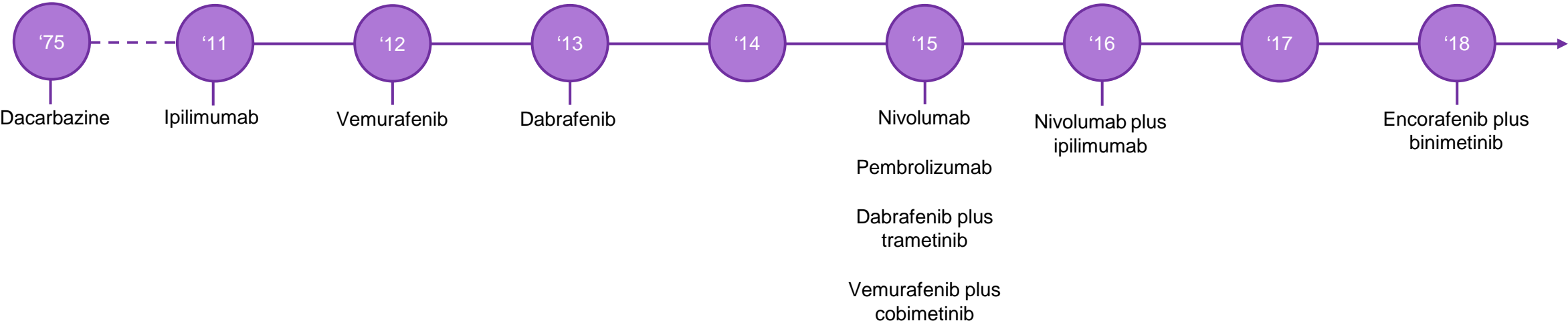

Supplement: Supplementary Figure S1 [file mmc1.pdf]

**Supplemental Figure A.** Division of the cost components stratified by cohort year

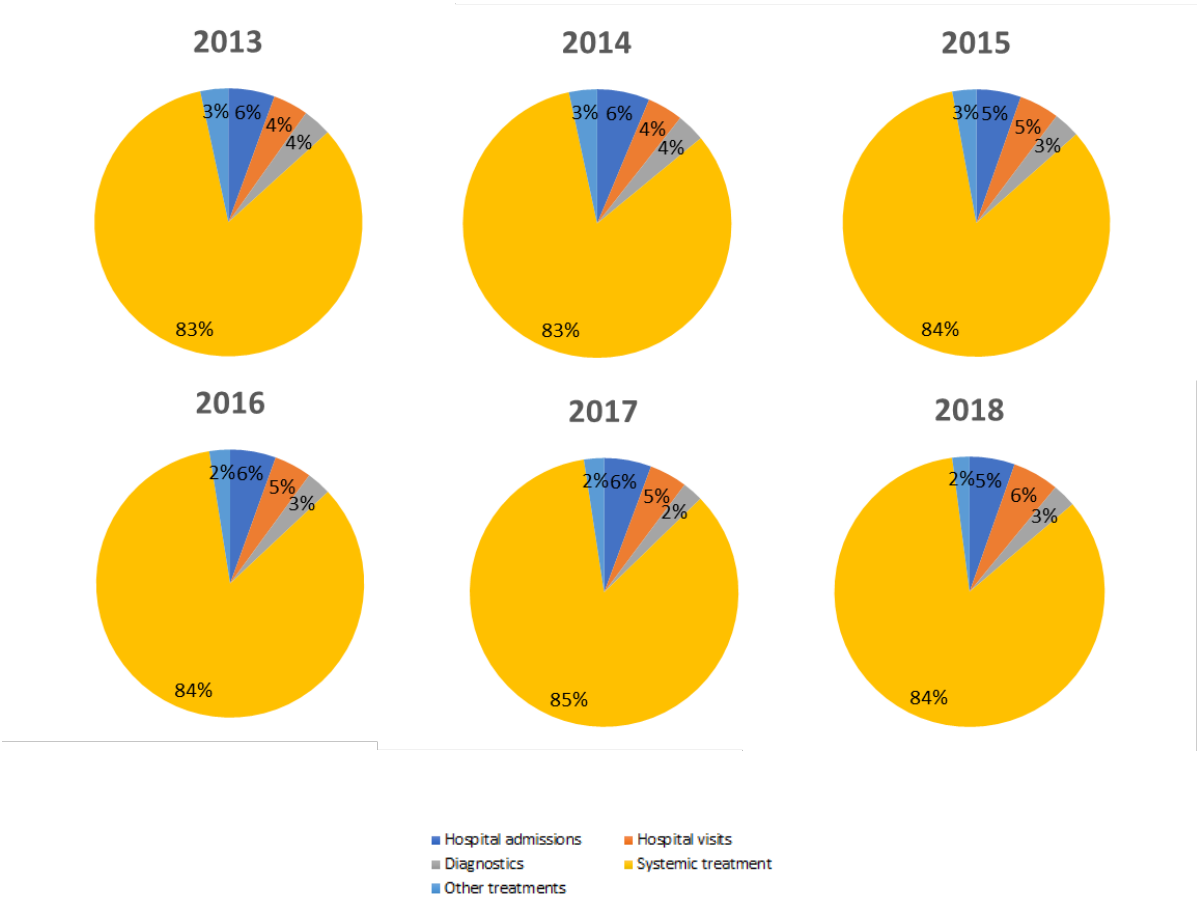

Supplement: Supplementary Figure S2 [file mmc2.pdf]
