# Supplementary material for: Trends in survival and costs in metastatic melanoma in the era of novel targeted and immunotherapeutic drugs
Source: ESMO Open. 2021 Nov 29;6(6):100320. doi: 10.1016/j.esmoop.2021.100320 (PMC8639434; doi:10.1016/j.esmoop.2021.100320)

Supplemental Figure B. Kaplan Meier overall survival patients diagnosed with stage IV melanoma between 2003 and 2010 (data of the Netherlands Cancer Registry)

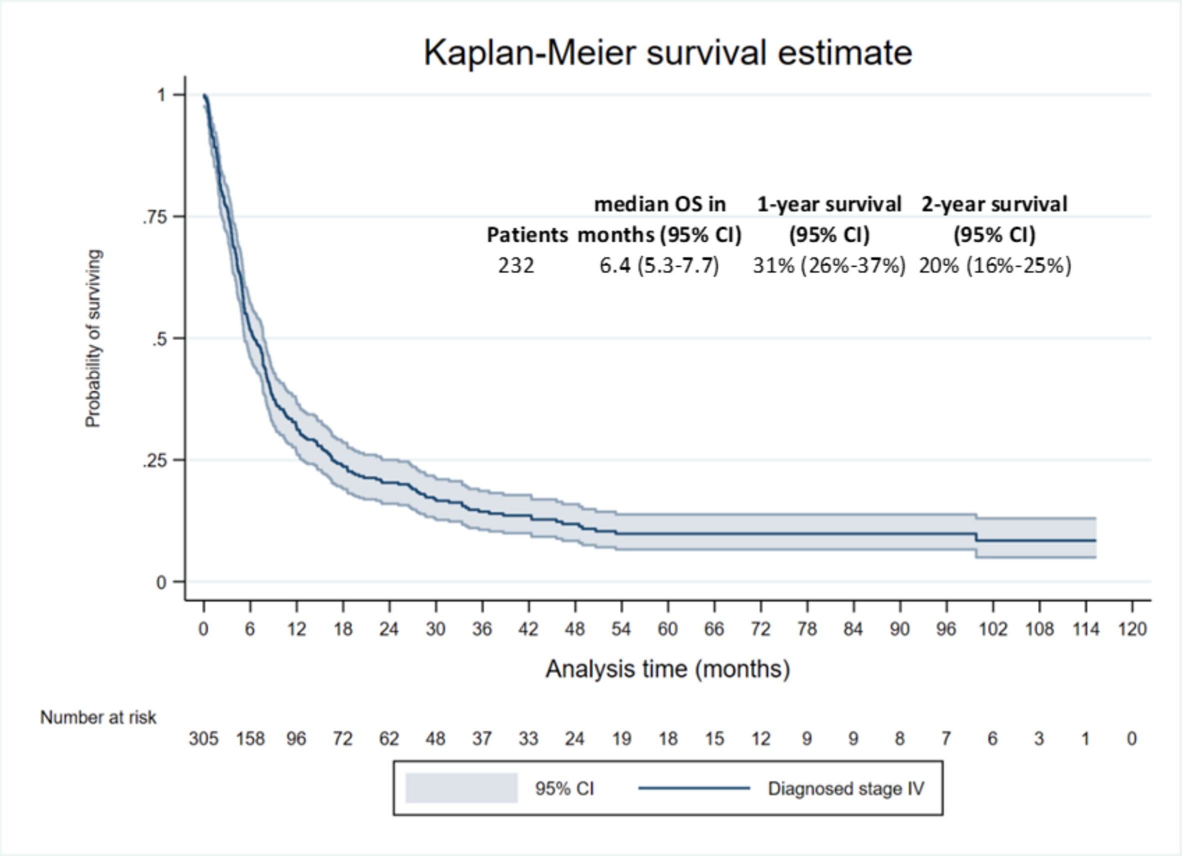

Supplement: Supplementary Figure S3 [file mmc3.pdf]
