# Supplementary material for: Trends in survival and costs in metastatic melanoma in the era of novel targeted and immunotherapeutic drugs
Source: ESMO Open. 2021 Nov 29;6(6):100320. doi: 10.1016/j.esmoop.2021.100320 (PMC8639434; doi:10.1016/j.esmoop.2021.100320)
Supplement: Supplementary Figure S4 [file mmc4.pdf]

Supplemental Figure C. Kaplan Meier estimates overall survival from start of metastatic disease stratified by synchronous and metachronous metastatic disease

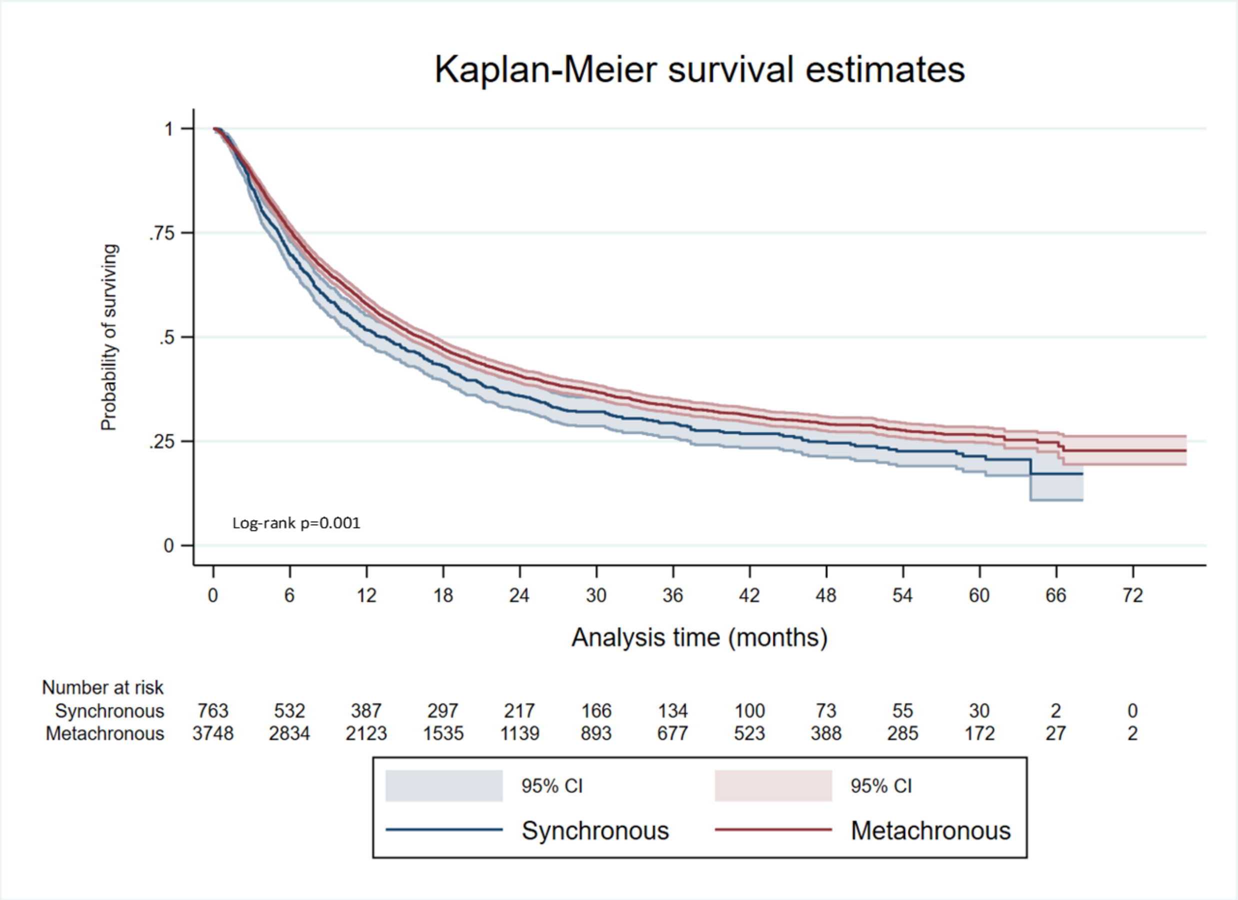

To allow comparison with historical data of the Netherlands Cancer Registry in which patients are recorded according to their initial diagnosis, we stratified all patients based on their diagnoses. For the DMTR this implied that patients were either diagnosed with synchronous metastatic disease or metachronous stage IV disease. All patients diagnosed with melanoma more than 2 months before the first visit in a melanoma center and patients with M0 disease were categorized as metachronous metastatic disease.
