# Supplementary material for: Trends in survival and costs in metastatic melanoma in the era of novel targeted and immunotherapeutic drugs
Source: ESMO Open. 2021 Nov 29;6(6):100320. doi: 10.1016/j.esmoop.2021.100320 (PMC8639434; doi:10.1016/j.esmoop.2021.100320)
Supplement: Supplementary Table S1 [file mmc5.pdf]

Supplemental Table A. Unit costs

Resource use	Unit costs	Source
**Hospital admissions**		Dutch costing manual[a]
Inpatient hospital day	€ 495.30	
Intensive care unit day	€ 1,234.08	
**Hospital visits**		Dutch costing manual
Outpatient visit	€ 94.69	
Daycare treatment	€ 287.19	
**Genetic testing**		Dutch Healthcare Authority[b]
Gene mutation testing	€ 929.25	
**Medical imaging**		Dutch Healthcare Authority
Computed tomography (CT) scan	€ 154.21	
Magnetic resonance imaging (MRI) scan	€ 285.91	
Positron emission tomography (PET)/CT scan	€ 1,069.76	
**Radiotherapy and hyperthermia**		Dutch Healthcare Authority
Radiotherapy short course (≤ 6 sessions)	€ 2,034.13	
Radiotherapy standard course (> 6 sessions)	€ 4,840.38	
Hyperthermia	€ 10,877.17	
Radiofrequency ablation	€ 1,490.84	
**Surgery**		Dutch Healthcare Authority
Excision	€ 95.65	
Lymph node dissection	€ 1,734.62	
Metastasectomy[d]	€ 2999.07 - € 6239.07	
**Systemic treatment**		Z-index[c]
Binimetinib		
tablet 15 mg	€ 34.09	
Cobimetinib		
tablet 20 mg	€ 86.89	
Dacarbazine		
vial 500mg	€ 46.33	
vial 1000 mg	€ 87.15	
Dabrafenib		
capsula 50 mg	€ 35.53	
capsula 75 mg	€ 52.16	
Encorafenib		
capsula 50 mg	€ 24.41	
capsula 75 mg	€ 36.05	
Investigational drugs[e]	€ 0.00	
Ipilimumab		
vial 50 mg	€ 4,250.00	
vial 200 mg	€ 17,000.00	
Nivolumab		
vial 40 mg	€ 405.03	
vial 100 mg	€ 1,012.56	
vial 240 mg	€ 2,430.15	
Pembrolizumab		
vial 50 mg	€ 1,312.18	
vial 100 mg	€ 2,624.37	
Temozolomide		
capsula 5 mg	€ 2.60	
capsula 20 mg	€ 4.80	
capsula 100 mg	€ 17.40	
capsula 140 mg	€ 24.00	
capsula 180 mg	€ 30.40	
capsula 250 mg	€ 40.20	
Trametinib		
tablet 0.5 mg	€ 54.19	
tablet 2 mg	€ 203.81	
Vemurafenib 240mg		
tablet 240 mg	€ 30.70	

[a] *Reference prices published in the Dutch costing manual by Hakkaart et al.*

[b] *Tariffs published by the Dutch Healthcare Authority*

[c] *Dutch national drug database, excluding VAT*

[d] *Ranging from €2999.07 for soft tissue metastases to €6239.07 for pancreatic metastases*

[e] *Costs of investigational drugs were set at zero if the drug was given in a blinded trial or if the drug was not approved for metastatic melanoma in The Netherlands at the time of this study*
